# #patientstoo – Professional sexual misconduct by healthcare professionals towards patients: a representative study

**DOI:** 10.1017/S2045796021000378

**Published:** 2021-06-21

**Authors:** V. Clemens, E. Brähler, J. M. Fegert

**Affiliations:** 1 Department for Child and Adolescent Psychiatry/Psychotherapy, University of Ulm, Ulm, Germany; 2 Department for Psychosomatic Medicine and Psychotherapy, University Medical Center of Johannes Gutenberg University of Mainz, Mainz, Germany; 3 Institute of Medical Psychology, Medical School, University of Leipzig, Leipzig, Germany

**Keywords:** Professional sexual misconduct, sexual contact, sexual harassment, healthcare professionals, professional-patient-relationship

## Abstract

**Aims:**

Sexual border violations are a severe problem in the healthcare system. Studies using non-probability samples indicate a high prevalence of professional sexual misconduct (PSM) towards patients. However, valid prevalence rates are lacking.

**Methods:**

We did a cross-sectional, observational study in Germany from February to April 2020. By different sampling steps, a probability sample of the German population above the age of 14 was generated. The final sample consisted 2503 persons (50.2% female, mean age: 49.5 years). Participants were asked about sexual contacts with and sexual harassment by healthcare professionals. Using descriptive statistics, prevalence rates of PSM were estimated.

**Results:**

PSM was reported by 56 (4.5%) female and 17 (1.4%) male participants. In detail, 28 (2.2%) female and 10 (0.8%) male participants reported sexual contacts with healthcare professionals. One third of these sexual contacts took place before the age of 18 and one third against the will of the patients. 40 (3.2%) female and 8 (0.6%) male participants reported unnecessary physical examinations, 31 (2.5%) female and 7 (0.6%) male participants reported sexual harassment. The majority of perpetrators were male.

**Conclusions:**

Our data provide an important first insight into the prevalence of PSM by healthcare professionals towards patients in a representative sample. Results suggest a high prevalence of PSM in the general population of Germany. Preventive measures to increase awareness of PSM and concepts for protection of patients are needed.

## Introduction

The relationship between patients and healthcare professionals is characterised by a high level of trust. Often, patients not only entrust their bodies to healthcare professionals, but also intimate information about their privacy and psyche. Disparities of power and knowledge and a high degree of vulnerability of patients add to the delicacy of this relationship. Therefore, sexual conduct between a healthcare professional and a patient can never be consensual (Federation of State Physician Health Program, 2019).

Already in the Hippocratic Oath it is laid down: ‘Into whatever houses I enter, I will go into them for the benefit of the sick, and will abstain from every voluntary act of mischief and corruption; and, further from the seduction of females or males, of freemen and slaves.’ (The Editors of Encyclopaedia Britannica, 1998) Thus, the exclusion of sexually motivated contact with patients has been existing for over 2000 years. Professional medical entities uniformly condemn sexual contact between healthcare professionals and their patients (Federation of State Medical Boards (FSMB), 2020).

However, the exploitation of power gaps, such as in a professional−patient relationship, is often not understood as such – especially by the person who is in the position of power. At the same time the vulnerability of the other is often misinterpreted as voluntary action (Alaggia and Wang, 2020). Adding to the problem, people who experience sexual misconduct often do not disclose, due to feelings of guilt, shame, fear and powerlessness (Ahrens *et al*., 2010). Strong hierarchies and relationships characterised by high intimacy such as those found in the healthcare system encourage sexual border violations (National Academies of Sciences and Medicine, 2018; Schröttle *et al*., 2019).

Partly as a result of the #metoo debate, studies on sexual harassment of healthcare professionals – by superiors, colleagues or patients – have been increasing in the last years (Hu *et al*., 2019; Jenner *et al*., 2019; Liu *et al*., 2019; Thurston *et al*., 2019; Vargas *et al*., 2020). However, studies assessing professional sexual misconduct (PSM) towards patients systematically are missing. This is surprising, as existing data – mainly based on analyses of disciplinary actions (Dehlendorf and Wolfe, 1998; Arora *et al*., 2014; Liu *et al*., 2015; Melo *et al*., 2019; Teegardin and Norder, 2019) and self-report information obtained through anonymous surveys among physicians (Gartrell *et al*., 1992; Coverdale *et al*., 1995; Bayer *et al*., 1996; Leusink and Mokkink, 2004) – point towards a high prevalence of PSM towards patients (Sansone and Sansone, 2009). However, as these analyses target physicians, no prevalence for PSM in the general public can be given.

Therefore, the present study aims to systematically assess PSM by healthcare professionals towards patients in a probability sample of the general population in order to provide valid prevalence estimates.

## Methods

### Study design

A representative sample of the German population was randomly generated by a research institute (USUMA, Berlin). Data collection took place between February and April 2020. A systematic area sampling was used (ADM F2F Sampling Frame), based on the municipal classification of the Federal Republic of Germany, covering the entire inhabited area of Germany. On the basis of these data, around 53 000 areas in Germany are delimited electronically, containing an average of around 700 private households in each area. These areas are first layered regionally according to districts into a total of around 1500 regional layers and then divided into 128 ‘networks’. One such network was then used as sampling frame, containing 258 single sample points proportionate to the distribution of private households in Germany.

In the second selection stage, private households to be surveyed at each sample point were systematically selected with a random route procedure. Households of every third residence in a randomly selected street were invited to participate in the study. As the third step of selection, in multi-person households, a kish-selection grid was used to ensure random participation. Participants had to be at least 14 years old and have sufficient German language skills to participate.

Individuals who agreed to participate were given information about the study and provided informed consent. In the case of minors, participants gave informed assent with informed consent being provided by their caregivers. Participants were told that the study was about psychological health and well-being. Responses were anonymous. In a first step, socio-demographic information was obtained in an interview-format by the research staff. Then, the researcher handed out a copy of the questionnaire and a sealable envelope. The completed questionnaires were linked to the respondent's demographic data, but did not contain name, address, or any other identifying information.

In all, 5668 households were initially contacted, 2503 people filled out the survey (response rate: 44.1%). The main reasons for non-participation were refusal of the selected household to provide information (23.5%), failure to contact persons in the household after four attempts (13.4%) and refusal of the target person to participate (13.2%). Data on the final sample are given in Table 1.

**Table 1.** Sample characteristics

Gender (female)	1256 (50.2)
Age (*M*, s.d.)	49.53 (17.51)
Living with partner (*n*, (%))	418 (16.7)
German citizenship (*n*, (%))	2409 (96.2)
Family status (*n*, (%))	
Married, living together	1108 (44.3)
Married living apart	55 (2.2)
Single	734 (29.3)
Divorced	375 (15.0)
Widowed	226 (9.0)
Not stated	5 (0.2)
Religion (*n*, (%))	
Protestant	934 (37.3)
Catholic	729 (29.1)
Muslim	60 (2.4)
Other (Jewish, Buddhist, Hindu)	44 (1.8)
No Religious affiliation	620 (24.8)
Not stated	116 (4.6)
Highest level of education (*n*, (%))	
School student	37 (1.5)
No graduation	52 (2.1)
‘Hauptschulabschluss’ (year 9, lower secondary school certificate)	702 (28.0)
‘Mittlere Reife’ (year 10, lower secondary school certificate)	805 (32.2)
Graduated from Polytechnical Highschool	195 (7.8)
Graduated from technical college with no accreditation	112 (4.5)
A-Level Certificate	340 (13.6)
University degree	258 (10.3)
Not stated or other	2 (0.1)
Equalised disposable income (*M*, s.d.)	
Below poverty threshold (<1.000)	268 (10.8)
Above poverty threshold (>1.000)	2250 (89.2)

Presented as number (*n*) or mean value (*M*) and standard deviation (s.d.) and (%). *N* = 2503.

### Ethical approval

The study was conducted in accordance with the Declaration of Helsinki, and was approved by the Ethics Committee of the Medical Department of the University of Leipzig.

### Measures

Socio-demographic questions used for this study included age, gender and socioeconomic status. To assess the prevalence of PSM, participants were asked whether they had ever had sexual contact with a healthcare professional, whether a healthcare professional had ever examined them physically without necessity in sexual intention, and whether they were ever sexually harassed by a healthcare professional while in a treatment relationship with him/her. In detail, the following questions were used “Have you ever had sexual contact (sexually motivated physical contact, e.g. “groping”, oral, vaginal or anal sexual intercourse) with a healthcare professional (physician r, nurse, psychotherapist, alternative practitioner, physiotherapist, other health care professional) while you were in a treatment relationship with him/her?”, “Did you ever have the impression that healthcare professionals (physician, nurse, psychotherapist, alternative practitioner, physiotherapist, other health care professionals) performed medical measures with sexual intent that would not have been necessary due to your complaints/illness?” and “Have you ever been sexually harassed by a healthcare professional (physician, nurse, psychotherapist, alternative practitioner, physiotherapist, other health professional) while you were in a treatment relationship with him/her?”. Directly after each of these three questions, the following items were asked for each: “If yes, how old were you at the time?” (Multiple answers possible)”, possible answers “Younger than 18 years” and “18 years or older”. Next, for each of the 3 types of sexual misconduct, we stated separately “If there were multiple sexual contacts, please relate the following 3 questions to the first time.”/ “If you had the impression several times in your life that unnecessary medical procedures were performed with sexual intent, please relate the following 3 questions to the first time.” / “If you were harassed several times in your life by healthcare professionals, please relate the following 3 questions to the first time.” and asked 1) “What was the gender of the healthcare professional?”, 2) “What was the profession of the health care professional?” and 3) “In which setting did the sexual contact take place?”. In the case of sexual contact with healthcare professional was affirmed, the participants were asked “If you were 18 or older at the time of contact, did you consent to sexual contact?”. Sexual contact under the age of 18 years was defined as involuntarily per se due to the age of the patient.

If more than one experience of sexual misconduct was reported, participants were asked to refer to the first experience for further details.

### Statistical analyses

All statistical analyses were performed with SPSS, version 21. Prevalence rates were determined by descriptive analyses. Only valid cases were evaluated. For the question at what age the experiences took place, only participants aged 18 and over were interviewed. The number of cases included is shown for each analysis.

## Results

### Prevalence of PSM

PSM was reported by 56 (4.5%) of female and 17 (1.4%) of male participants. In detail, 28 (2.2%) female and 10 (0.8%) male participants reported sexual contacts with healthcare professionals. Half of the sexual contacts over the age of 18 happened against the will of the patient. 40 (3.2%) female and 8 (0.6%) male participants reported unnecessary physical examinations, 31 (2.5%) female and 7 (0.6%) male participants reported sexual harassment by healthcare professionals. The majority of experiences of PSM were reported by participants who were 60 years old or younger (see Table 2).

**Table 2.** Prevalence of PSM

	Sexual contact	Unnecessary physical examination	Sexual harrassment	Any type
Gender				
Female	28 (2.2)	40 (3.2)	31 (2.5)	56 (4.5)
Male	10 (0.8)	8 (0.6)	7 (0.6)	17 (1.4)
*p value*	*0.01*	*<0.001*	*<0.001*	*<0.001*
Age group				
⩽40 years	16 (2.0)	23 (2.9)	14 (1.8)	30 (3.8)
41–60 years	18 (1.9)	20 (2.1)	22 (2.3)	35 (3.7)
⩾61 years	4 (0.5)	5 (0.7)	2 (0.)	8 (0.3)
*p value*	*0.032*	*0.006*	*0.003*	*0.002*
Overall	38 (1.5)	48 (1.6)	38 (1.5)	76 (2.9)
Not consensual^a^	12 (44.4)			

Presented as *n* (%). Sexual contact: *n* = 2501, unnecessary physical examination: *n* = 2500, sexual harassment: *n* = 2498.

a Was only asked of participants who were over 18 years old at the time of sexual contact (*n* = 27).

### Age of patients at the time of experiencing PSM

**Fig. 1.**
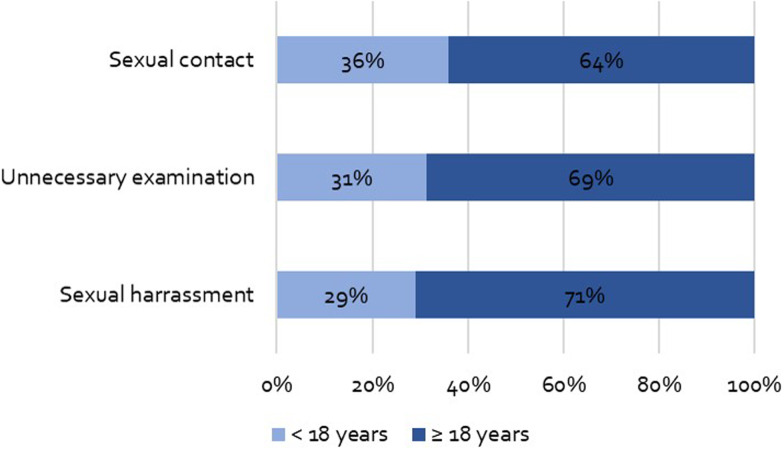
Age of patients.

A high proportion of patients were minors during the experience of PSM. In detail, 14 participants (36%) of the participants who reported sexual contact, reported having been under 18 years of age at the time of sexual contact with healthcare professionals. 15 participants (31% of the participants who reported harassment) were minors at the time of the examinations. 11 participants (29% of those who reported unnecessary physical examinations) were minors at the time of the examinations (see Fig. 1).

### Gender of healthcare professionals

**Fig. 2.**
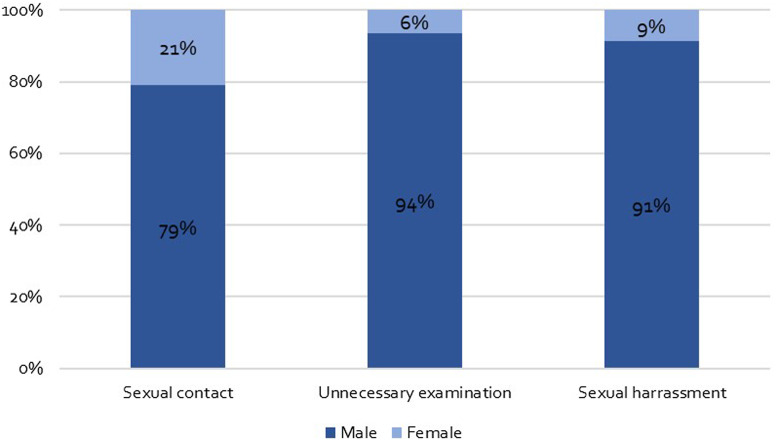
Gender of perpetrator.

Men committed the majority of PSM. In detail, 30 participants reported that the sexual contacts took place with male healthcare professionals (corresponding to 79% of the reported sexual contacts). A total of 32 participants reported sexual harassment by male healthcare professionals (91% of all reported sexual harassment) and 44 participants who have experienced unnecessary physical examinations reported that they were carried out by males (corresponding to 94% of all reported unnecessary physical examinations) (see Fig. 2).

### Setting of PSM

**Fig. 3.**
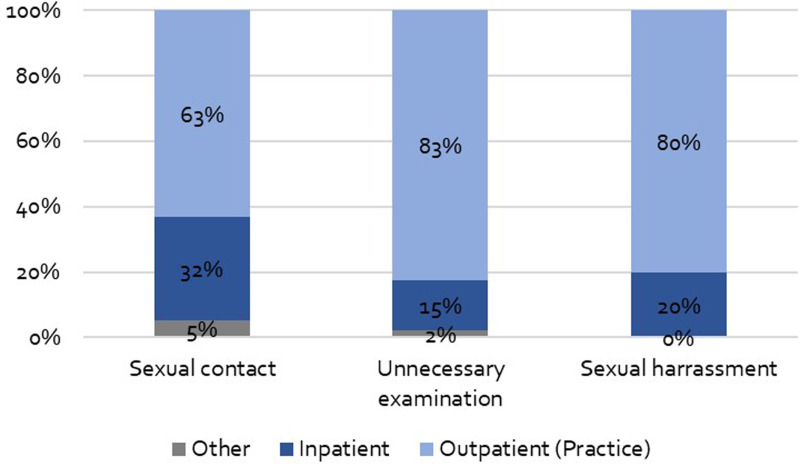
Setting of PSM.

The majority of sexual misconduct occurred in the outpatient setting. In detail, 24 participants (63% of the participants who reported having sexual contact with a healthcare professional) stated that it took place in an outpatient setting. 28 participants (80% of those affected) reported to have experienced sexual harassment in the outpatient setting. 38 participants (83% of those who reported unnecessary physical examinations) stated that it took place in the outpatient setting (see Fig. 3).

### Profession of healthcare professionals

Physicians were most frequently named as the perpetrators of PSM. In detail, 13 (36%) of participants who reported sexual contacts said that took place with physicians and 14 (40%) of participants who reported sexual harassment as well as 27 (60%) of participants who reported unnecessary physical examinations said that they were carried out by physicians. Besides physicians, sexual contacts happened most frequently with nurses, named by 8 (22%) of affected participants, and psychotherapists, named by 7 (19%) of affected participants. Besides physicians, sexual harassment was most frequently experienced from nurses and physiotherapists, reported by 7 participants each (20% of those affected). Beside physicians, unnecessary physical examinations were carried out most frequently by physiotherapists, reported by 7 participants (16% of affected participants) and nurses, named by 5 affected participants (11%) (see Table 3).

**Table 3.** Profession of healthcare professionals

	Sexual contact		Unnecessary physical examination		Sexual harassment	
Physician	13	(36)	27	(60)	14	(40)
Psychotherapist	7	(19)	3	(7)	5	(14)
Nursing staff	8	(22)	5	(11)	7	(20)
Physiotherapist	3	(8)	7	(16)	7	(20)
Alternative practitioner	2	(6)	1	(2)	1	(3)
Paramedic	2	(6)	1	(2)	1	(3)
Other	1	(3)	1	(2)	0	(0)

Sexual contact: *n* = 36, unnecessary physical examination: *n* = 45, sexual harassment *n* = 35. Presented as *n* (%) of the participants who reported the respective form of PSM.

## Discussion

The present study is the first to systematically assess PSM by healthcare professionals towards patients based on a probability sample of the general population in Germany. Our results indicate high rates of PSM. 4.5% of female and 1.4% of male participants have reported any form of sexual misconduct by healthcare professionals. Extrapolated to the population of Germany over 14 years of age (Statista, 2020*b*), 2 000 000 subjects in Germany have experienced PSM by healthcare professionals.

In Germany, for healthcare professionals it is illegal to have sexual contact with a patient during a treatment relationship regarding paragraph 174 (c) of the German Criminal Code. Sexual contact with subjects below the age of 18 by taking advantage of an exploitative situation is prohibited by paragraph 182 of the German Criminal Code and characterised as sexual abuse of juveniles.

Anonymous surveys of physicians estimate that 3.3–14.5% of physicians have performed PSM (Sansone and Sansone, 2009). An online survey of the US Federation of State Medical Boards (FSMB) indicated that 18% of Americans have had an interaction with a physician who they believe was acting unethically, unprofessionally, or providing substandard care (Federation of State Medical Boards (FSMB), 2019). However, as this was a non-probability sample, bias cannot be excluded, e.g. due to oversampling of affected subjects. Thus, no valid prevalence rates for the population can be estimated from that study. Oversampling of affected subjects may also be one reason for the significantly higher prevalence of PSM in the FSMB study compared to our data. Additionally, in the FSMB study, not only sexual but also other forms of misconduct were included.

In a previous analysis of the German population, 0.8% of those who were treated in hospitals or rehabilitation centres during childhood or adolescence experienced sexual abuse by nursing staff during their stay (Clemens *et al*., 2019). The present study confirms a high proportion of minors who experience sexual abuse during treatment. However, in this study, besides nursing staff, also other healthcare professions and other settings besides inpatient stays were assessed. This may explain the higher proportion of affected participants seen in the present study.

Sexual contact was reported by 2.2% of female and 0.4% of male participants. Even though a sexual contact between healthcare professionals and patients can never be consensual due to the significant power gap and the vulnerable position of the patient, we have asked affected participants whether these were consensual to assess how patients interpret the sexual contact. Nearly half of the participants who reported sexual contact after the age of 18 answered that the sexual contact was not consensual – indicating a high number of participants not wanting sexual contact. This is highly alarming. On the other hand, more than 50% of those who reported sexual contact with healthcare professionals above the age of 18 stated that the contact was consensual – indicating that they were not aware of the special character of a relationship between patient and healthcare professional. Depending on the character of the treatment, some patients may not have been aware of their vulnerable position. As we have not assessed details of the relationship between patient and healthcare professionals, it may be the case that some knew the healthcare professional personally before treatment start. Other patients may aim to avoid feelings of fear and powerlessness by negotiating involuntary contact. The high prevalence rates of PSM are in strong contrast to the low crime statistics of less than 600 cases per year in Germany (Statista, 2020*a*). This suggests that the problem of PSM has been clearly underestimated in Germany to date. The FSBMB online survey confirms low rates of reporting and, moreover, the majority of patients did not know where to report conduct or other complaints (Federation of State Medical Boards (FSMB), 2019).

Our data show that the vast majority of healthcare professionals committing PSM were male, while significantly more female participants reported to have experienced PSM. This is in line with the results of other studies indicating males as main perpetrators of PSM (Sansone and Sansone, 2009; DuBois *et al*., 2019). A study from the US on attitudes of physicians from different specialties towards a number of personal relationships with patients showed that male physicians were more likely to advocate sexual contact with patients than female physicians (Regan *et al*., 2010).

PSM took place mostly in outpatient settings. In the outpatient setting, patients are more often alone with healthcare professionals, while in inpatient setting, e.g. fellow patients or colleagues are often present when patients are contacted. This reduced social control in outpatient settings may facilitate PSM.

All forms of PSM were most frequently committed by physicians. This seems logical as nearly everyone has contact with physicians regularly but not with other healthcare professionals such as physiotherapists or others. Besides, sexual contact occurred conspicuously frequently with psychotherapists. Against the background that only 7–10% of the German population have undergone psychotherapy in their life (Astrid *et al*., 2013), this indicates a relatively very high prevalence of sexual contact in this area. During psychotherapy, there is often a long-lasting relationship between the therapist and the patient, which is accompanied by a high degree of closeness and intimacy. This could increase the risk of sexual contact (Luepker, 1999) by increasing needs and fantasies while weakening objectivity and thus control (American Psychiatric Association, 2001). Our results are in line with other studies showing highest rates of sexual misconduct in psychiatrists (Brooks *et al*., 2012; Melo *et al*., 2019; Schröttle *et al*., 2019). Physiotherapy, on the other hand, is much more common. The 12-month prevalence of physiotherapy is 20% in Germany (Rommel and Prütz, 2017). Still, physiotherapists were the 4th most frequent named profession, potentially indicating that physical contact may facilitate PSM. The 12-month prevalence of alternative medicine in Germany is high and ranges between 40 and 62% (Linde *et al*., 2014), although this includes both, alternative medicine performed by physicians and alternative practitioners. However, although relative rates may be lower compared to physicians and nurses, our results indicate that PSM is a problem among all assessed professions.

Sexual harassment has health consequences for those affected and is associated with poorer physical and mental health (Thurston *et al*., 2019). The results of our study indicate a high proportion of sexual contact before the age of 18. The experience of sexual abuse in childhood/adolescence can have profound influence on the rest of one's life. Consequences can include several physical illnesses (Irish *et al*., 2010), psychological problems and social impairments (Ferrara *et al*., 2016). Additionally, PSM may significantly violate trust in healthcare professionals and institutions, with possibly additional harmful consequences for the health of affected subjects due to avoidance and noncompliance.

The aim of this study was to assess the prevalence of sexual PSM by healthcare professionals towards patients. However, it should be mentioned that sexual border violations also exist in the other direction: many healthcare professionals report of border violations by patients (Liu *et al*., 2019; Vargas *et al*., 2020). Rather, the high prevalence of sexual border violations from both sides shows that measures are needed to protect both, patients and healthcare professionals, and setting clear boundaries in the relationship between healthcare professionals and patients favours both.

A central limitation of the present study is that although the total number of participants is high, the number of participants in the sub-categories is sometimes limited. To enable a more precise characterisation of patients' experiences, a study with more participants would be necessary. More detailed information would also be important for targeted prevention. This includes the context of PSM and e.g. whether healthcare professionals and patients knew each other before the treatment. The high number of underage patients should also be subject to further investigations. Another limitation is that from the patient's point of view, it is not always possible to assess with certainty whether or not a physical examination is necessary. Nevertheless, this item gives an important indication about how patients assessed the situation. The U.S. Equal Employment Opportunity Commission defines sexual harassment as ‘unwelcome sexual advances, requests for sexual favors, and other verbal or physical conduct of a sexual nature […]’ (Gallo *et al*., 2018). In the German language, the term ‘sexual harassment’ is not limited to a workplace setting. However, as we gave no definition of sexual harassment in our questionnaire, the understanding of what sexual harassment comprises may vary between participants. Moreover, we focused on PSM during a treatment relationship. Yet, it is important to point out that also after treatment relationships, the power gap between healthcare professionals and former patients and the vulnerable position of the former patient may last. This is particular the case after psychotherapy, why it is has to be pointed out that professional responsibilities continued after treatment (Appelbaum and Jorgenson, 1991; Shavit and Bucky, 2004). The German federal chamber of psychotherapists prohibit any private contacts with former patients in the 1st year after end of treatment (Bundespsychotherapeutenkammer, 2007).

Overall, despite these limitations, the here presented data provide an important first insight into the prevalence of PSM by healthcare professionals towards patients in the general population. Our data point towards a high prevalence of PSM in Germany despite its illegality. Against the background of the potential harmful consequences for survivors of PSM, there is an urgent need for greater awareness of the problem of PSM in healthcare professionals. The FSMB Workgroup on PSM formulated best practice recommendations for effectively addressing and preventing sexual misconduct (Federation of State Medical Boards (FSMB), 2020). Proposed measures include a culture of support that does not tolerate any harassment. This social control seems to be central in prevention of PSM and, on the basis of our results, to be less effective in outpatient settings. Consequently, there is a need to take different workplace settings into account if preventive measures are planned. Individual risk assessments of each health care provider may be central in this context. This was recently demanded by the Federal Joint Committee (G-BA), the highest decision-making body of the joint self-government of physicians, dentists, hospitals and health insurance funds in Germany and will be mandatory by the end of 2021. This risk assessment may include an assessment where patients are alone with individual healthcare professionals, especially during examinations with lots of physical contact, examinations of intimate body zones and long-lasting treatment relationships characterised by high intimacy, but also of situations where patients feel unsafe or uncomfortable. However, considering sexual border violations of patients towards healthcare professionals, such risk assessments shall also include an assessment of situations where healthcare professionals feel unsafe. Further measures proposed by the FSMB comprise systematic handling of complaints, mandatory reporting of complaints and a standardised handling of investigations. One key action is the implementation of PSM, the reasons its illegality and possible consequences into education and training. Exemplarily, the University of Toronto ensures adequate education about appropriate physician-patient and teacher−learner boundaries by implementation of a course concerning physician−patient sexual misconduct and teacher−learner mistreatment and harassment (Robinson and Stewart, 1996). The high proportion of sexual contacts before the age of 18 in our survey is highly concerning. Sexual abuse of minors by healthcare professions seem to be a serious child protection problem, specific measures to protect minors in medical institutions are needed.

## Data Availability

The datasets generated during the current study are not publicly available due to conditions on participant consent.
